# Altered nucleocytoplasmic proteome and transcriptome distributions in an *in vitro* model of amyotrophic lateral sclerosis

**DOI:** 10.1371/journal.pone.0176462

**Published:** 2017-04-28

**Authors:** Jee-Eun Kim, Yoon Ho Hong, Jin Young Kim, Gye Sun Jeon, Jung Hee Jung, Byung-Nam Yoon, Sung-Yeon Son, Kwang-Woo Lee, Jong-Il Kim, Jung-Joon Sung

**Affiliations:** 1 Department of Neurology, Seoul Medical Center, Seoul, Republic of Korea; 2 Department of Neurology, Seoul National University Seoul Metropolitan Government Boramae Medical Center, Seoul, Republic of Korea; 3 Division of Mass Spectrometry Research, Korea Basic Science Institute, Daejun, Korea; 4 Department of Neurology, Seoul National University Hospital, Seoul, Korea; 5 Macrogen Inc., Seoul, Korea; 6 Department of Neurology, Inha University Hospital, Incheon, Korea; 7 Department of Neurology, Eulji University Hospital, Daejun, Korea; 8 Department of Biochemistry and Molecular Biology, Seoul National University College of Medicine, Seoul, Korea; University of Toronto, CANADA

## Abstract

Aberrant nucleocytoplasmic localization of proteins has been implicated in many neurodegenerative diseases. Evidence suggests that cytoplasmic mislocalization of nuclear proteins such as transactive response DNA-binding protein 43 (TDP-43) and fused in sarcoma (FUS) may be associated with neurotoxicity in amyotrophic lateral sclerosis (ALS) and frontotemporal lobar degeneration. This study investigated the changes in nucleocytoplasmic distributions of the proteome and transcriptome in an in vitro model of ALS. After subcellular fractionation of motor neuron-like cell lines expressing wild-type or G93A mutant hSOD1, quantitative mass spectrometry and next-generation RNA sequencing (RNA-seq) were performed for the nuclear and cytoplasmic compartments. A subset of the results was validated via immunoblotting. A total of 1,925 proteins were identified in either the nuclear or cytoplasmic fractions, and 32% of these proteins were quantified in both fractions. The nucleocytoplasmic distribution of 37 proteins was significantly changed in mutant cells with nuclear and cytoplasmic shifts in 13 and 24 proteins, respectively (p<0.05). The proteins shifted towards the nucleus were enriched regarding pathways of RNA transport and processing (Dhx9, Fmr1, Srsf3, Srsf6, Tra2b), whereas protein folding (Cct5, Cct7, Cct8), aminoacyl-tRNA biosynthesis (Farsb, Nars, Txnrd1), synaptic vesicle cycle (Cltc, Nsf), Wnt signalling (Cltc, Plcb3, Plec, Psmd3, Ruvbl1) and Hippo signalling (Camk2d, Plcb3, Ruvbl1) pathways were over-represented in the proteins shifted to the cytoplasm. A weak correlation between the changes in protein and mRNA levels was found only in the nucleus, where mRNA was relatively abundant in mutant cells. This study provides a comprehensive dataset of the nucleocytoplasmic distribution of the proteome and transcriptome in an in vitro model of ALS. An integrated analysis of the nucleocytoplasmic distribution of the proteome and transcriptome demonstrated multiple candidate pathways including RNA processing/transport and protein synthesis and folding that may be relevant to the pathomechanism of ALS.

## Introduction

Amyotrophic lateral sclerosis (ALS) is a relentlessly progressive fatal neurodegenerative disease that affects motor neurons [1]. While a complete understanding of ALS pathogenesis remains incomplete, multiple mechanisms have been proposed including glutamate toxicity, oxidative stress, protein misfolding, altered axonal transport, mitochondrial dysfunction, and defects in RNA processing [1].

It has been increasingly recognized that protein mislocalization may play an important role in ALS/FTLD pathology. Of note, TAR DNA-binding protein 43 (TDP-43) and fused in sarcoma (FUS), which are both RNA-binding nuclear proteins, are mislocalized from their normal nuclear compartment to the cytoplasm and form cytoplasmic aggregations [2–4]. Nucleocytoplasmic transport defects may link the nuclear depletion and cytoplasmic aggregation of these proteins [5]. In addition, recent studies have also suggested that the C9orf72 hexanucleotide repeat expansion (HRE), the most common causative mutation in familial ALS, exerts toxicity by disrupting nucleocytoplasmic transport [6–8]. Furthermore, several of the latest studies using different HRE disease models have identified the disease-modifying genes that encode elements of nuclear pore complex and nuclear RNA export/nuclear protein import machinery [6–8]. Proteins associated with nucleocytoplasmic transport such as RanGAP1 were also found to be mislocalized in autopsied brain tissues and in induced pluripotent stem cells derived from ALS patients with the C9orf72 mutation [7].

With the advent of quantitative proteomics, large-scale proteomic analyses are now possible [9]. Recent developments in chemical peptide labelling with isobaric tags such as tandem mass tag (TMT) and iTRAQ have allowed for the expression levels of thousands of proteins to be compared across complex samples [10,11]. Subcellular fractionations followed by high-throughput techniques also provide an opportunity to investigate the subcellular distribution of the proteome and mislocalization in pathological conditions (“spatial proteomics”). Here, we employed TMT tagging to investigate proteome-wide nucleocytoplasmic changes in an in vitro model of ALS by employing NSC34 motor neuron-like cell lines expressing wild-type or G93A mutant human SOD1 (hSOD1). The exact pathomechanisms of SOD1 mutations, the second most common causative gene for familial ALS, are not fully understood [12], and little is known regarding proteome-wide changes in the nucleocytoplasmic distribution. RNA sequencing (RNA-seq) was also performed to compare the changes in the proteomes and transcriptomes of the nucleus and cytoplasm of the in vitro ALS model.

## Materials and methods

### Cell culture and subcellular fractionation

The NSC34 motor neuron-like cell lines (kindly provided by H Ryu, Korea Institute of Science and Technology, Seoul, Korea) was stably transfected with the pCI-neo expression vector containing wild-type or G93A mutant hSOD1 as described previously [13,14]. Cells were maintained in Dulbecco’s modified Eagle’s medium (JBI, Korea), with 10% heat-inactivated fetal bovine serum (Gibco, Grand Island, NY, USA), and 100 U/ml penicillin-100 mg/ml streptomycin (Gibco, Grand Island, NY, USA). Cells were maintained in a humidified incubator at 37°C under 5% CO_2_.

For subcellular fractionation, we used the commercially available NE-PER nuclear and cytoplasmic extraction reagent (Thermo Scientific, USA). Briefly, the wild-type and mutant NSC-34 cell pellets were washed with phosphate-buffered saline (PBS) and separated by centrifugation. The supernatant was discarded, and the pellet was resuspended in cytoplasmic extraction reagent (CER) I by vigorous vortexing. Next, CER II was added, and the tube was vortexed for 10 seconds and then centrifuged for 4 minutes at 16,000 x g. The supernatant was transferred as a cytoplasmic extraction. The insoluble fraction that contained the nuclear component was suspended in ice-cold nuclear extraction reagent (NER) and vortexed for 15 seconds every 10 minutes for a total of 40 minutes. After centrifugation, the supernatant was prepared for the nuclear fraction. The extraction was stored at -80°C until use.

### Materials for TMT

The following materials were used for TMT labeling: formic acid, urea, tris(2-carboxyethyl)phosphine (TCEP), iodoacetamide (IAA) (Sigma-Aldrich, St. Louis, MO, USA), a Labesix Plex reagents kit (Thermo Scientific, No. 90064), and an HPLC-grade acetonitrile (Burdick and Jackson, (Muskegon, MI, USA). A Milli Q system (Millipore, Molsheim, France) was used for water purification.

### Sample preparation for proteome analysis

One hundred micrograms of the protein extract achieved from each cell fraction was dissolved in 45 μL of 200 mM triethylammonium bicarbonate (TEAB) buffer (pH 8) containing 8 M urea. Next, 500 mM TCEP was added at room temperature and mixed for 60 min. Then, the mixture was alkylated for 60 min with 500 mM IAA in a dark environment at room temperature. The samples were desalted using a membrane filter of 10 KMW and then were dissolved in 200 mM TEAB (pH 8) buffer to a final protein concentration of 1 μg/μL. Each concentration of protein was calculated using a bicinchoninic acid (BCA) assay (Thermo Scientific), as described in the manufacturer’s protocol. Sequencing-grade trypsin (Promega, Madison, WI, USA) and the proteins in the TEAB buffer were mixed in a 1:20 (wt/wt) ratio and incubated overnight at 37°C [15]. We used three experimental replicates for each wild-type and G93A mutant hSOD1-transfected NSC34 cells. Three replicate sets of cells were grown and harvested with each set from a separate passage of single stable cell line. After subcellular fractionation, the samples from wild-type hSOD1-transfected cells were individually labelled using MT-126, 128, and 130, and those from G93A mutant hSOD1-transfected cells were labelled using TMT-127, 129, and 131, according to the manufacturer’s protocol. An aqueous hydroxylamine solution (5% w/v) was blended to finish the reaction. Finally, proteins from six samples were pooled, dried with a speed-vacuum, and melted in 0.1% formic acid with 50 μL water for liquid chromatography-tandem mass spectrometry (LC-MS/MS).

### 2D-LC-MS/MS

The 2D-LC-MS/MS system, made up of a nanoACQUITY UltraPerformance LC System (Waters, USA) and an LTQ Orbitrap Elite mass spectrometer (Thermo Scientific, USA) with a nano-electrospray source, were used to analyse the TMT-labelled samples [16]. A strong intensity cation exchange (5 μm, 3 cm) column was located before the C_18_ trap column (180 μm i.d., 20 mm length, 5 μm particle size; Waters). For each run, peptide solutions were used in 5 μL aliquots. Then, peptides were deranged by a salt gradient, introduced over an autosampler loop, from the strong intensity cation exchange phase into the C_18_ phase, and desalted at a flow rate of 4μL/min for 10 min. Next, the trapped peptides were detached on a 200 mm homemade microcapillary column made up of C_18_ (Aqua; particle size 3μm), filled into a 100μm silica tube with a 5μm orifice id.

A ten-step salt gradient was applied using 3 μL of 0, 25, 50, 100, 250, and 500 mM ammonium acetate (0.1% formic acid in 5% acetonitrile) and then adding 4, 5, 9 and an additional 9 μL of 500 mM ammonium acetate (0.1% formic acid in 30% acetonitrile). The mobile phase A was composed of 0% acetonitrile and 0.1% formic acid, and phase B was composed of 100% acetonitrile and 0.1% formic acid. The LC gradient was initiated with 5% B for 1 min, increased to 20% B over 5 min, 50% B over 90 min, 95% B over 1 min, and then maintained at 95% B for 3 min and 5% B for an additional 5 min [17,18]. Before the next run, the column was re-balanced with 5% B for 15 min, and a 2.0 kV voltage was applied to induce an electrospray. The LTQ Orbitrap Elite was regulated based on a data-dependent approach during the chromatographic separation. The MS data were obtained by collecting five data-dependent collision induced dissociation-high energy collision dissociation (CID-HCD) dual MS/MS scans for each full scan with the following parameters: HCD scans and full scans obtained in Orbitrap at resolutions of 60,000 and 15,000 by two-microscan averaging, CID scans obtained in the LTQ by two-microscan averaging, 35% of normalized collision energy (NCE) in CID, 45% of NCE in HCD, and ±1 Da isolation window. Fragmented ions were expelled for 60 sec. Each parent ion was initially fragmented using a CID and followed by an HCD in a CID-HCD dual scan [15].

### Protein identification and quantification

An International Protein Index (IPI) mouse database (IPI.MOUSE. 7.26.2012) was used for the MS/MS spectra analysis, following the software analysis protocols. To calculate the false discovery rate (FDR), conversed sequences of all proteins were attached to the database. ProLucid was employed to classify the peptides with the following parameters as a precursor: a mass error of 25 ppm and a mass error distribution of fragment ions in 600 ppm [19]. Trypsin was chosen as the enzyme with 2 potential missed cleavage sites. Lysine and N-terminus TMT modification and cysteine carbamidomethylation were selected as static modifications. Methionine oxidation was selected as a variable modification. To ensure better peptide identification and quantification, the tandem CID and HCD MS spectra from the identical precursor ions were co-analysed using software [20]. In-house software, in which reporter ions from the HCD spectrum were inserted into the CID spectrum with identical precursor ions as the earlier scan, was used. Reporter ions were pulled from small windows (±20 ppm) near their anticipated m/z in the HCD spectrum. DTASelect (The Scripps Research Institute, USA) was used to filter and sort the output data to build the protein list. Two or more peptides were entered to identify the proteins, and ‘less than 0.01’ was set for the false positive rate [21]. Peptide quantification was conducted using Census (Version 1.98, Integrated Proteomics, USA) (S1 and S2 Tables). The peptide intensities were log2 transformed, and quantile normalization was performed. Peptides that were not identified in either the nuclear or cytoplasmic fraction were excluded. Peptide abundances were then “rolled up” to the protein level using the R-rollup method implemented in DanteR [22] (S3 Table). Peptides that were mapped to multiple proteins were included in the normalization procedure but excluded from the protein quantification step.

### RNA-seq

Three sets of NSC34 cells (transfected with wild-type or G93A mutant hSOD1) were grown and harvested with each set from a separate passage of single cell line. Following subcellular fractionation, transcriptomes of 12 samples were analysed by RNA-seq (Macrogen Inc. Seoul, Korea), as described previously. Briefly, 1 μg of the total RNA was analysed using the TruSeq RNA library kit to construct the cDNA libraries. The protocol included polyA-selected RNA extraction, RNA fragmentation, random hexamer primed reverse transcription and 100 nt paired-end sequencing using an Illumina HiSeq2000 (Illumina, San Diego, CA, USA). The libraries were quantified using quantitative real-time polymerase chain reaction (qPCR) according to the qPCR Quantification Protocol Guide. An Agilent Technologies 2100 Bioanalyzer was used for the qualification.

### Aligning RNA-Seq reads and quantification

We processed reads from the sequencer and aligned them to the *Mus musculus (mm9)* using Tophat v2.0.13 [23]. Tophat incorporates the Bowtie v2.2.3 algorithm to perform the alignment [24]. Tophat initially removes a portion of the reads based on the quality information accompanying each read before it maps reads to the reference genome. The reference genome sequence of *Mus musculus(mm9)* and annotation data were downloaded from the UCSC table browser (http://genome.uscs.edu). Gene annotation information was also used for running Tophat with the “-G” option. For the other Tophat parameters, the default options were used. Tophat allows multiple alignments per read (up to 20 by default) and a maximum of 2 mismatches when mapping the reads to the reference. After aligning the reads to the genome, Cufflinks v2.2.1 were used to assemble aligned reads into transcripts and to estimate their abundance [25]. To correct for sequence expression count bias, ‘—max-bundle-frags 50000000’ options were used. We also used the ‘-G’ option for making the best use of known gene annotation information. The default options were used for other parameters. The transcript counts in isoform level were calculated, and the relative transcript abundances were measured in fragments per kilobase of exon per million fragments mapped (FPKM) from Cufflinks.

We used FPKM as the expression level to analyse differentially expressed transcripts. The FPKM values were normalized by factors such as transcript length and total number of reads. During preprocessing, we performed data filtering, data transformation and between-sample normalization to filter DE transcripts. Transcripts with zero FPKMs more than one across all samples were excluded. To facilitate the statistical analysis with a balanced distribution, we added 1 to the FPKMs of the filtered data and transformed the data to log 2. The log-transformed data were then ranked in quantiles containing identical numbers by quantile normalization. We adjusted for batch effect using the ComBat algorithm (http://www.bu.edu/jlab/wp-assets/ComBat/Abstract.html).

To investigate mRNA splicing defects, we quantified intronic and exonic reads by using HTseq with an intersection-strict option [26]. Reads were counted as intron and exon reads, when they were uniquely mapped within introns and exons, respectively.

### Statistical analysis

The statistical analysis was conducted using R 3.0.0 (www.r-project.org, reference). For the proteins quantified in both nuclear and cytoplasmic fractions, the effects of the hSOD1 genotype (mutant vs. wild-type) and the subcellular compartment (nucleus vs. cytoplasm) were analysed using a two-way ANOVA model. The interaction term, i.e., hSOD1 genotype × subcellular compartment, was used to assess differences in nuclear cytoplasmic distributions between mutant and wild-type cells. The correlation between the changes in the proteome and transcriptome was analysed in each subcellular compartment using Pearson correlation coefficient. Protein IPI identifiers were mapped to corresponding gene identifiers. When a single gene was associated with multiple proteins, the protein with the largest abundance value was selected for the gene. Statistical significance was set at p = 0.05.

### Bioinformatics annotation

An integrated pathway clusters analysis of the identified proteins that exhibited significant alterations in their total amount or nuclear cytoplasmic distribution in the mutant cells was performed using TargetMine (http://targetmine.mizuguchilab.org/). Furthermore, the identified proteins were also categorized by universal gene ontology (GO) terms using the DAVID tool (version 6.8, http://david.abcc.ncifcrf.gov/). The adjusted p value, <0.05, was defined as the threshold.

### Validation of the proteome

The identified proteins with statistically significant changes were validated by Western blotting. Cell lysates were prepared from the proposed experimental conditions, subjected to sodium dodecyl sulphate polyacrylamide gel electrophoresis. Immunoblots were probed with the following antibodies: Atp5b, Cct5, Cct8, Hist1h1a, Hist1h1b, LaminB, β-actin (Santa Cruz Biotechnology, Santa Cruz, CA), Hist1h1e (Abcam, Cambridge, MA), and hSOD-1 (Cell Signaling, Danvers, MA) followed by treatment with the appropriate secondary antibodies conjugated to horseradish peroxidase (Bethyl Laboratories, Montgomery, TX). SuperSignal West Pico substrate (Pierce-Thermo, Northumberland, UK) and ImageQuant LAS 4000 (GE Healthcare Bio-Sciences, Pittsburgh, PA) were employed to visualize immunoreactive bands. β-actin was used as the loading control.

## Results

### Validation of subcellular fractionation

**Fig 1:**
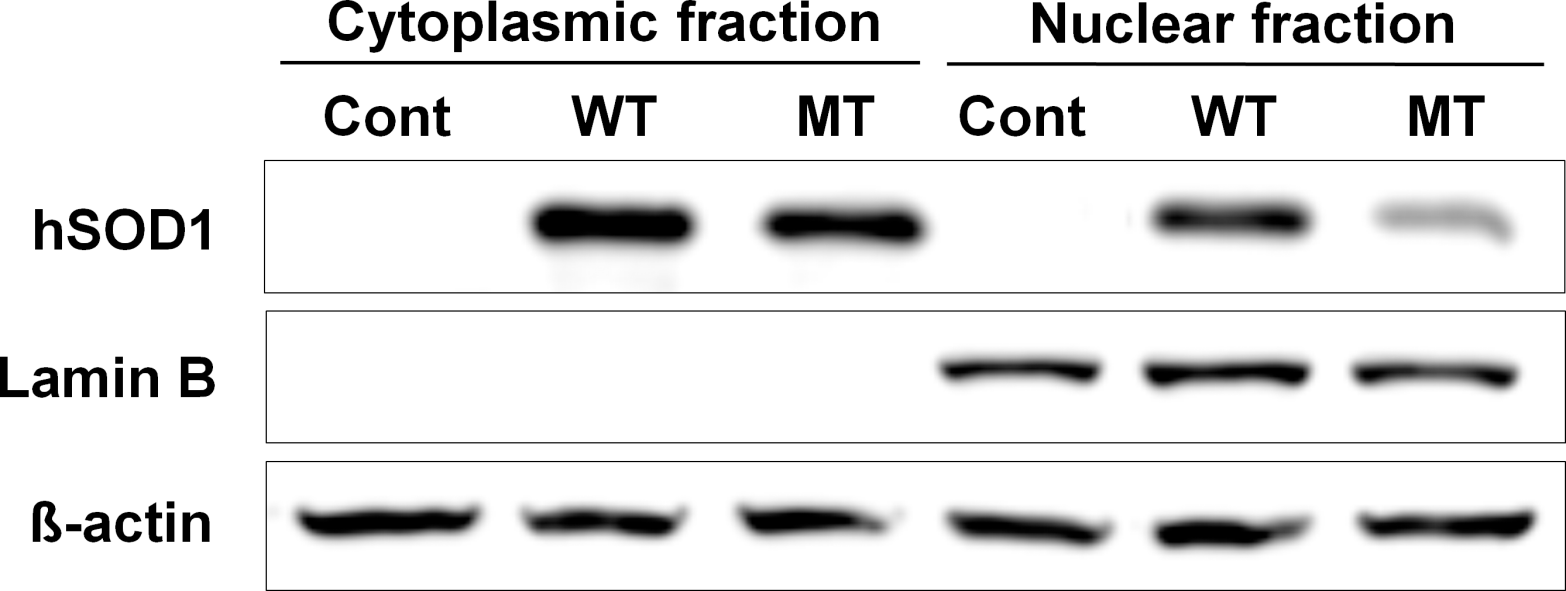
Western blots of marker proteins from the nuclear and cytoplasmic fractions of the NSC34 cells expressing the wild-type (WT) or mutant human SOD1 (G93A). Lamin B, a nuclear protein marker, was exclusively expressed in the nuclear fraction. Actin was used as a loading control to confirm equal protein loading. Cont, Control; MT, mutant cells; WT, wild-type cells.

The NSC34 cell lines stably transfected with pCI-neo expression vector containing wild-type or G93A mutant hSOD1 were fractionated into nuclear and cytoplasmic fractions by using the NE-PER kit. Mock transfected NSC34 cells were served as control group. To validate subcellular fractionation preparation, the isolated nuclear and cytoplasmic fractions were immunoblotted for lamin B (a nuclear marker). Immunoblots showed a clear separation of the two subcellular fractions (Fig 1). The western blot also demonstrated that wild-type hSOD1 was present at similar level in both nuclear and cytoplasmic fractions, whereas mutant hSOD1 was mainly cytoplasmic. This may be explained by the formation of insoluble high molecular weight species of mutant hSOD1 that prevent the diffusion of the protein across the nuclear membrane. Indeed, we observed the presence of larger amount of hSOD1 in the insoluble fraction of NSC34 cells expressing G93A mutant hSOD1 (data not shown).

To further investigate the validity of subcellular fractionation procedure, we analyzed distribution of organelle marker proteins by using pRoloc data which provides 1,305 marker proteins across 55 different organelles [27]. Among these marker proteins, 65 proteins were identified exclusively in the nuclear fraction, whereas 69 proteins were identified exclusively in the cytoplasmic fraction. We next investigated the organelle membership of these proteins. As illustrated in S1A Fig, the proteins that are exclusively identified in nuclear fraction were mostly labeled as nuclear organelles such as nucleus, chromatin, and nucleolus. In contrast, the most representative organelles of the proteins exclusively identified in cytoplasmic fraction were proteasome, cytoplasm, and mitochondria. As for the proteins quantified in both fractions, the principle component analysis revealed a distinct separation of protein abundance profiles between the samples from nuclear versus cytoplasmic fraction along the first principal component (S1B Fig). We further analyzed the abundance level of nucleus and cytoplasm marker proteins across 12 samples (6 nuclear and 6 cytoplasmic fractions). It was clearly demonstrated that the levels of nucleus marker proteins were significantly higher in nuclear fraction, and that the levels of cytoplasm marker proteins were significantly higher in cytoplasmic fraction (S1C Fig). Given together, these results support the validity of subcellular fractionation procedure used in the present study.

### Protein quantification and analysis of nucleocytoplasmic distribution

**Fig 2:**
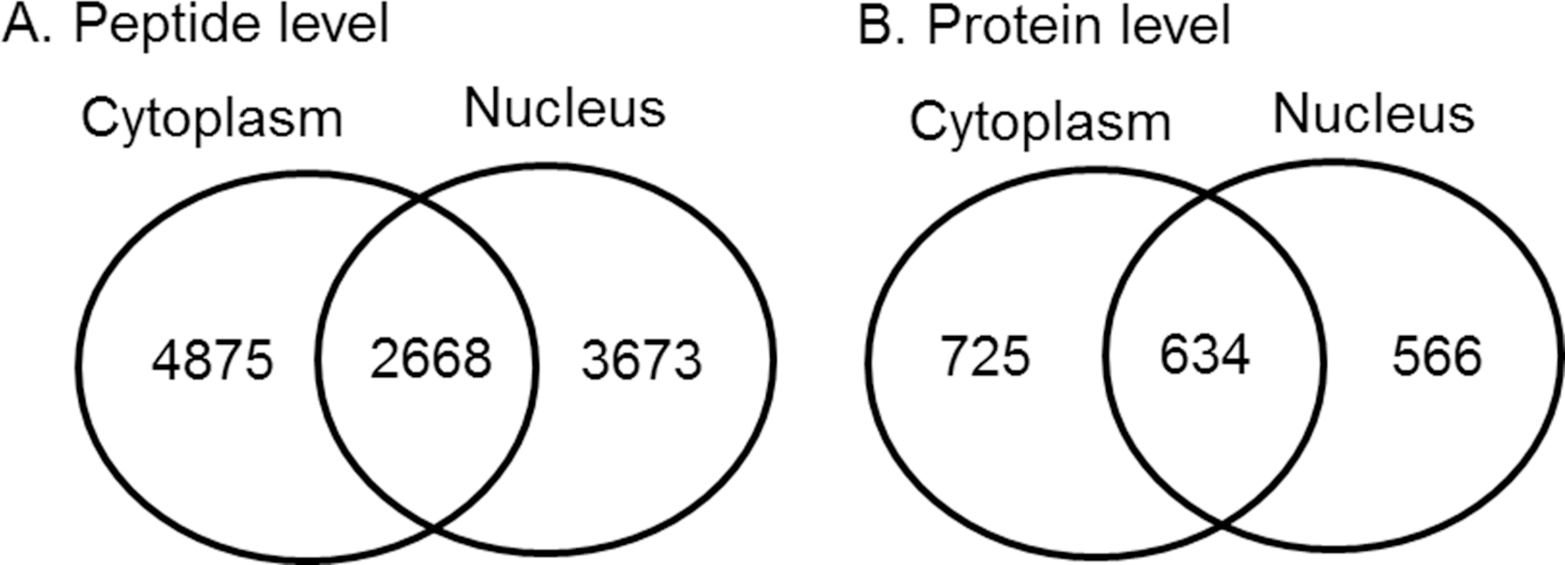
Venn diagrams showing the peptides (A) and proteins (B) quantified in the cytoplasmic and nuclear fractions of NSC34 lines stably transfected with wild-type or G93A mutant human SOD1.

**Fig 3:**
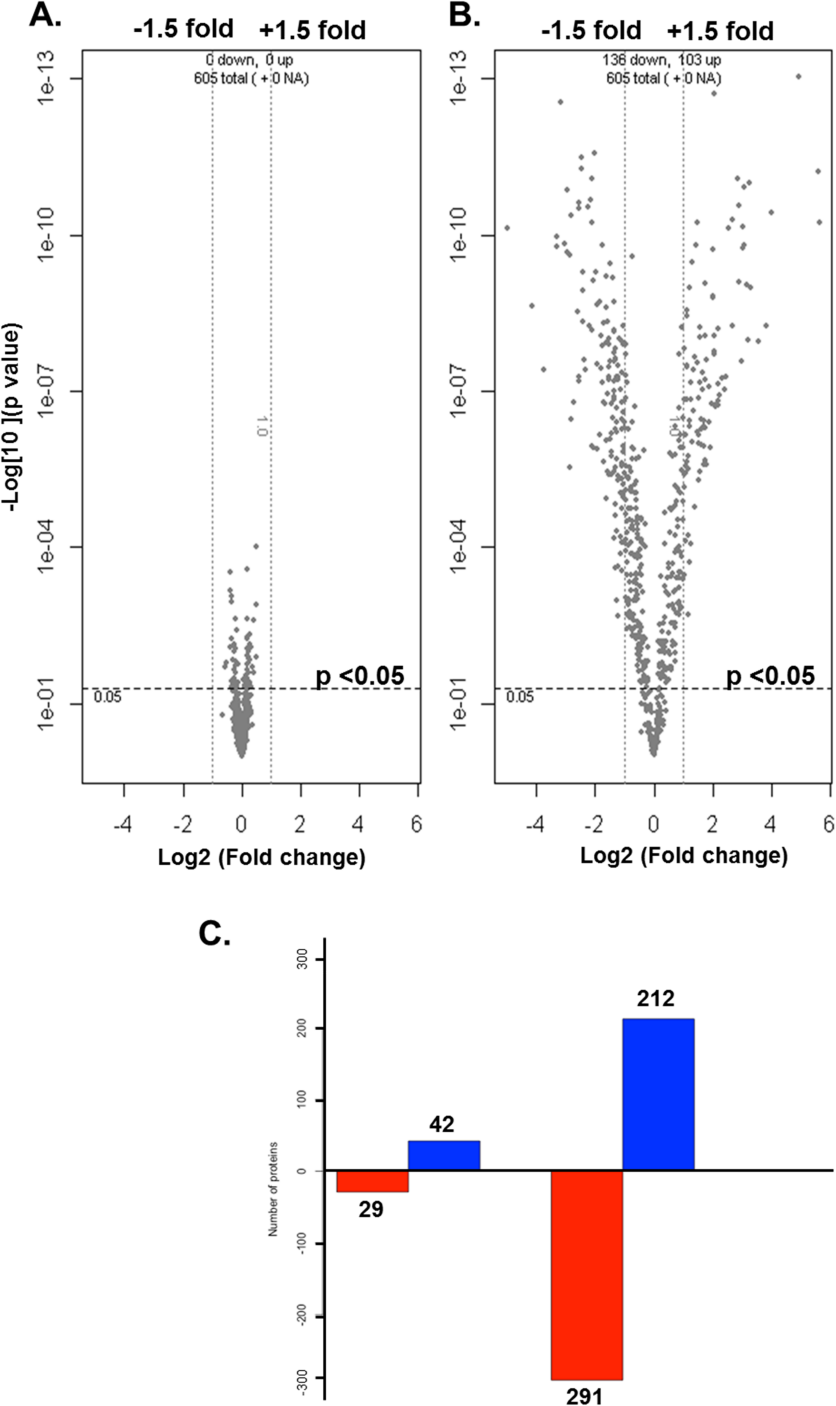
**Volcano plots showing the global changes of protein abundance in wild-type vs. mutant cells (A) and in the nucleus vs. cytoplasm (B).** The log2-fold changes in mutant vs. wild type and in the nucleus vs. cytoplasm are represented on the x-axis. The y-axis shows the negative log10-transformed raw p-values of two-way ANOVA tests. Bar plots (C) showing the number of proteins that are significantly down-regulated (in red) or up-regulated (in blue) in mutant cells compared to wild-type cells (left) and in the nucleus compared to the cytoplasm (right).

**Fig 4:**
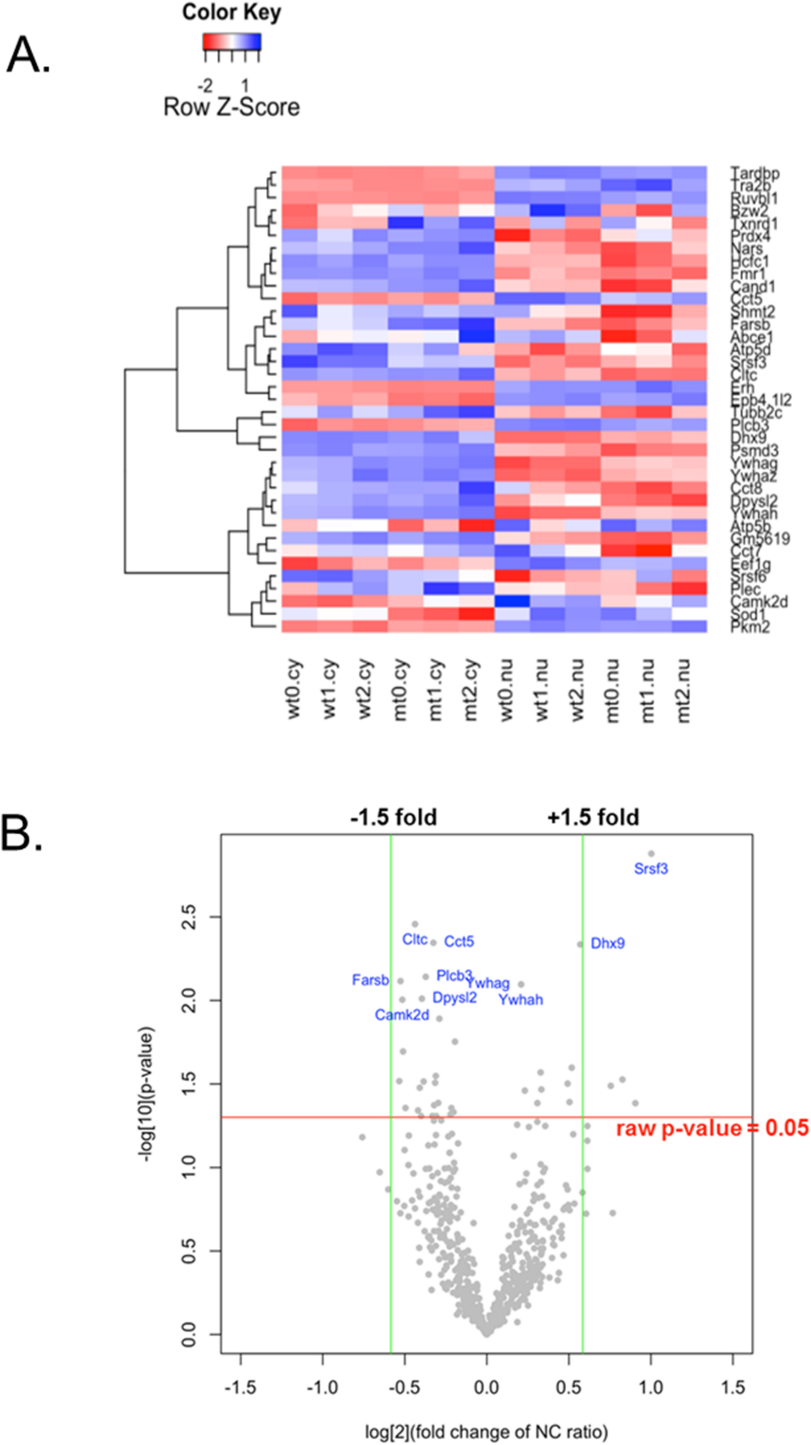
Alteration of proteome nucleocytoplasmic distribution. Heat map (A) representing color-coded abundance and a hierarchical cluster of 37 differentially localized proteins for biological triplicate samples of wild-type cytoplasmic, mutant cytoplasmic, wild-type nuclear, and mutant nuclear fractions (two-way ANOVA, raw p-values for the genotype × compartment interaction effect <0.05). The red represents low abundance, and blue represents high abundance. In the volcano plot (B), the estimated log2-fold change of the nucleocytoplasmic (NC) ratio in mutant versus wild-type cells is represented on the x-axis, and the negative log10-transformed raw p-values are shown on the y-axis.

Proteins were extracted from the nuclear and cytoplasmic fractions of NSC34 cells stably transfected with wild-type hSOD1 and G93A mutant hSOD1. After TMT-labeling, the samples were mixed and analysed by mass spectrometry (LC-MS/MS). A total of 1,359 proteins were identified from 7,543 peptides (unique in 93.8%) in the cytoplasm, and 1,200 proteins from 6,341 peptides (unique in 92.7%) in the nucleus. The nucleocytoplasmic memberships of the identified peptides and proteins are presented in Fig 2. Quantitative analysis of nucleocytoplasmic distribution was performed on 634 proteins identified in both the nuclear and cytoplasmic fractions. We used a two-way ANOVA model in which the effects of SOD1 genotype (wild-type vs. mutant), subcellular compartment (nucleus vs. cytoplasm), and their interaction (genotype × compartment) were assessed. A significant genotype effect was found among a small fraction of the proteins with 42 up-regulated and 29 down-regulated in mutant cells (p-value <0.05) (Fig 3A and 3C, Tables 1 and 2). In contrast, a considerable number of proteins (79.3% of total) were predominant in either nucleus or cytoplasm, exhibiting a significant compartment effect (p-value <0.05) (Fig 3B and 3C). Changes of the nucleocytoplasmic distribution in mutant cells were found for 37 proteins with a significant genotype × compartment interaction in the two-way ANOVA model. Nuclear shift was found in 13 proteins, and cytoplasmic shift in 24 proteins. These differentially localized proteins and their nucleocytoplasmic abundance ratios are summarized in Table 3. The changes of nucleocytoplasmic distributions were also represented by a heatmap with hierarchical clustering and a volcano plot (Fig 4). Among the exclusively nuclear or cytoplasmic proteins, no protein was found to be significantly up- or down-regulated in mutant cells (Wilcoxon rank sum test, p>0.05 in all). In addition, no protein was found be exclusively nuclear (or cytoplasmic) in wild-type cells but cytoplasmic (or nuclear) in mutant cells.

**Table 1:** Differentially up-regulated proteins in NSC34 cells expressing the mutant human SOD1 (G93A) compared to wild-type SOD1.

IPI	Gene symbol	Log[2](MT/WT)	IPI	Gene symbol	Log[2](MT/WT)
IPI00758024.1	Prdx6	0.49	IPI00321734.7	Glo1	0.19
IPI00649135.3	Gstm1	0.49	IPI00134353.3	Nol3	0.19
IPI00115650.4	Cacybp	0.49	IPI00649406.1	Park7	0.19
IPI00331704.7	Eno2	0.43	IPI00227392.5	Ywhah	0.19
IPI00222759.3	Vat1l	0.39	IPI00230707.6	Ywhag	0.19
IPI00317309.5	Anxa5	0.35	IPI00626994.3	Ipo5	0.19
IPI00417165.3	Enah	0.34	IPI00230429.4	Kpna3	0.18
IPI00121427.1	S100a6	0.31	IPI00116498.1	Ywhaz	0.18
IPI00885558.1	Pdia3	0.29	IPI00132575.3	Cotl1	0.18
IPI00990246.1	Nme1	0.28	IPI00331556.5	Hspa4	0.17
IPI00411075.2	Pcbp3	0.27	IPI00760000.1	Ywhab	0.17
IPI00990529.1	Gstp1	0.27	IPI00660514.1	Dnajb6	0.17
IPI00461281.2	Nudcd2	0.25	IPI00131224.1	Tceb2	0.17
IPI00757109.3	Pcmt1	0.24	IPI00123342.4	Hyou1	0.16
IPI00762774.2	Eif3d	0.23	IPI00798527.1	Tnpo1	0.16
IPI00269662.1	Hnrnpa3	0.23	IPI00776252.1	Txnrd1	0.14
IPI00153728.1	Ddx19b	0.22	IPI00314153.4	Yars	0.14
IPI00339916.10	Eprs	0.21	IPI00111181.1	Vps35	0.14
IPI00116254.1	Prdx4	0.21	IPI00323357.3	Hspa8	0.11
IPI00759940.3	Fh1	0.2	IPI00116308.1	St13	0.1
IPI00122743.2	Dars	0.2	IPI00119057.1	Eif4e	0.06

Two-way ANOVA, genotype effect, p-value < 0.05.

**Table 2:** Differentially down-regulated proteins in NSC34 cells expressing the mutant human SOD1 (G93A) compared to wild-type SOD1.

IPI	Gene symbol	Log[2](MT/WT)	IPI	Gene symbol	Log[2](MT/WT)
IPI00130589.8	Sod1	-0.57	IPI00123624.8	2610301G19Rik	-0.24
IPI00474974.1	Dnmt1	-0.54	IPI00230133.5	Hist1h1b	-0.23
IPI00169870.6	Glt25d1	-0.4	IPI00223371.3	Rbm39	-0.23
IPI00228616.5	Hist1h1a	-0.39	IPI00337844.5	Ranbp2	-0.22
IPI00132352.2	2610029G23Rik	-0.39	IPI00515398.1	Myh10	-0.22
IPI00109813.1	Hnrnpa0	-0.39	IPI00318725.4	Rrs1	-0.22
IPI00223714.5	Hist1h1e	-0.36	IPI00330289.4	Epb4.1l2	-0.21
IPI00113141.1	Cs	-0.36	IPI00754963.2	Mest	-0.19
IPI00229535.2	Gtf2i	-0.35	IPI00312128.3	Trim28	-0.17
IPI00331361.2	Mybbp1a	-0.34	IPI00828543.3	Hcfc1	-0.17
IPI00331597.6	Hist1h1d	-0.33	IPI00133985.1	Ruvbl1	-0.15
IPI00673465.2	Cnot1	-0.31	IPI00281011.7	Marcksl1	-0.15
IPI00154054.1	Acat1	-0.28	IPI00622811.2	Ap2m1	-0.15
IPI00515654.2	Eef1d	-0.27	IPI00881287.1	Fkbp8	-0.12
IPI00226882.7	Sec61a1	-0.25			

Two-way ANOVA, genotype effect, p-value < 0.05.

**Table 3:** Differentially localized proteins with nuclear or cytoplasmic shifts in NSC34 cells expressing the mutant human SOD1 (G93A) compared to wild-type SOD1.

IPI	Gene symbol	Log_2_(N/C)_WT_	Log_2_(N/C)_MT_	IPI	Gene symbol	Log_2_(N/C)_WT_	Log_2_(N/C)_MT_
IPI00407130.4	Pkm2	2.17	-0.63	IPI00469268.5	Cct8	-0.32	-0.69
IPI00133985.1	Ruvbl1	2.14	-0.28	IPI00114375.2	Dpysl2	-0.35	-0.49
IPI00988949.1	Erh	1.86	0.49	IPI00896727.1	Cand1	-0.4	-0.56
IPI00311203.2	Plcb3	1.44	-0.57	IPI00918997.1	Nars	-0.42	-0.5
IPI00330289.4	Epb4.1l2	1.41	0.7	IPI00322828.2	Farsb	-0.45	-0.95
IPI00280967.3	Tardbp	1.39	-0.27	IPI00116254.1	Prdx4	-0.65	0.25
IPI00970572.1	Tra2b	1.25	0.56	IPI00310880.4	Srsf6	-0.68	0.75
IPI00116279.3	Cct5	0.9	-0.43	IPI00227392.5	Ywhah	-0.73	0.13
IPI00318841.4	Eef1g	0.89	-0.49	IPI00230707.6	Ywhag	-0.73	0.13
IPI00406790.9	Camk2d	0.62	-0.75	IPI00828543.3	Hcfc1	-0.84	-0.27
IPI00130589.8	Sod1	0.46	1.81	IPI00116498.1	Ywhaz	-0.87	0.17
IPI00387337.1	Bzw2	0.31	-0.47	IPI00227013.2	Fmr1	-0.87	-0.23
IPI00468481.2	Atp5b	0.26	1.24	IPI00648173.1	Cltc	-0.89	-0.57
IPI00322869.3	Abce1	0.14	-0.55	IPI00314439.4	Psmd3	-0.93	-0.32
IPI00331174.5	Cct7	0.1	-0.6	IPI00462453.5	Gm5619	-1.07	-0.56
IPI00776252.1	Txnrd1	0.05	-0.48	IPI00453777.2	Atp5d	-1.36	1.47
IPI00230061.3	Plec	-0.08	-0.33	IPI00339468.4	Dhx9	-1.72	0.72
IPI00454008.1	Shmt2	-0.17	-0.41	IPI00221826.1	Srsf3	-1.85	1.68
IPI00169463.1	Tubb2c	-0.29	-0.51				

N/C, nucleocytoplasmic ratio of protein abundance; WT, wild-type cells; MT, mutant cells. Two-way ANOVA, genotype × compartment interaction effect, p-value < 0.05.

### Pathway analysis

We first checked if there were any pathways enriched for the proteins quantified in both fractions by using TargetMine [28]. As illustrated in S2 Fig, over-represented were the pathways such as RNA transport, metabolism of proteins, Wnt signalling, protein processing in the endoplasmic reticulum, and cell cycle.

Next, to investigate functional biological processes enriched for the differentially expressed proteins, we performed gene enrichment and functional annotation analysis by using DAVID online software (version 6.8). Biological processes enriched for up-regulated and down-regulated proteins were summarized in Table 4 with corresponding genes and p-values.

**Table 4:** Gene ontology (GO) functional annotation terms (biological process) enriched for the differentially expressed proteins (down-/up-regulated) in mutant cells.

Biological process	Gene	P.value
Upregulation in mutant cells		
Protein folding	St13, Nudcd2, Pdia3, Dnajb6, Hspa8	1.8E-04
Cell redox homeostasis	Pdia3, Prdx6, Prdx4, Txnrd1	3.9E-04
NLS-bearing protein import into nucleus	Ipo5, Kpna3, Tnpo1	6.3E-04
Negative regulation of apoptotic process	Hyou1, Ywhah, Nol3, Hspa4, Glo1, Park7, Gstp1	0.0014
Negative regulation of cardiac muscle cell apoptotic process	Nol3, Pcmt1, Hspa8	0.0014
tRNA aminoacylation for protein translation	Yars, Dars, Eprs	0.0028
Protein targeting	Ywhag, Ywhaz, Ywhab	0.0031
Negative regulation of extrinsic apoptotic signaling pathway	Nol3, Park7, Gstp1	0.0042
Glutathione metabolic process	Gstm1, Glo1, Gstp1	0.0052
Translation	Eif3d, Yars, Eif4e, Dars, eprs	0.011
Negative regulation of cell death	Cacybp, Hspa4, Park7	0.013
Intracellular protein transport	Ywhah, Ipo5, Vps35, Tnpo1	1.45E-02
Downregulation in mutant cells		
Nucleosome assembly	Hist1h1a, Hist1h1b, Hist1h1d, Hist1h1e	3.4E-04
Negative regulation of transcription from RNA polymerase II promoter	Hist1h1e, Hist1h1d, Trim28, Hcfc1, Dnmt1, Cnot1	0.0023
Covalent chromatin modification	Trim28, Hcfc1, Dnmt1, Ruvbl1	0.0051
Regulation of transcription, DNA-templated	Gtf2i, Trim28, Hcfc1, Dnmt1, Cnot1, Rbm39, Ruvbl1, Eef1d, Mybbp1a	0.0071
Transcription, DNA-templated	Gtf2i, Trim28, Dnmt1, Cnot1, Rbm39, Ruvbl1, Eef1d, Mybbp1a	0.0092

GO, gene ontology; DNA, deoxyribonucleic acid; NLS, nuclear localization signal; RNA, ribonucleic acid; tRNA, transfer ribonucleic acid.

**Fig 5:**
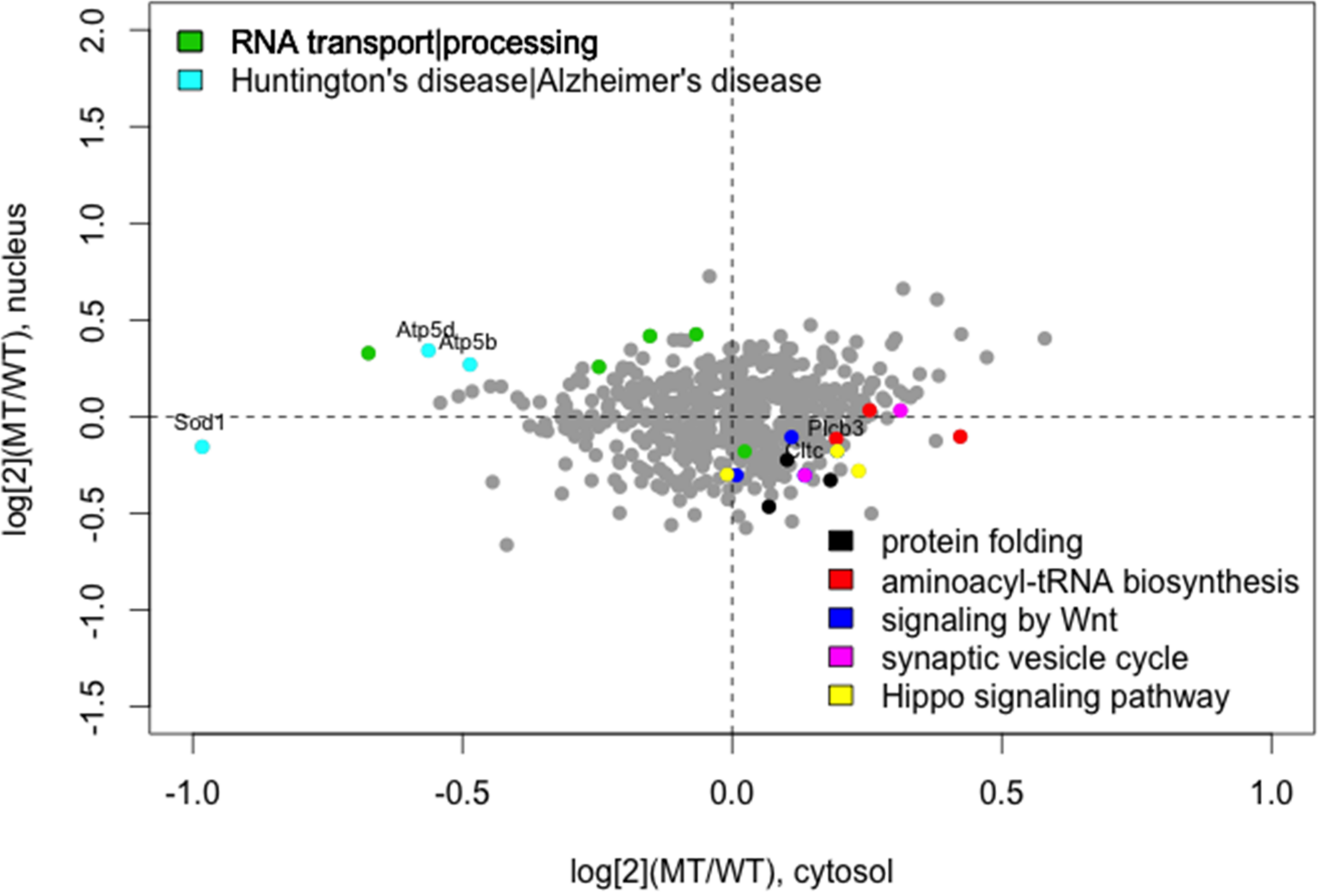
Scatter plot of the changes in protein abundance in the cytoplasmic (x-axis) and nuclear (y-axis) fractions. The changes are expressed as the log ratio of protein abundance in mutant vs. wild-type cells. Color-coded dots represent differentially localized proteins. Different colors for different enriched pathways. The cytoplasmic shift in mutant cells is represented in the right lower quadrant, and nuclear shift is represented in the left upper quadrant. Gene symbols are annotated in the plot.

To investigate over-represented pathways across differentially localized proteins, we performed pathway enrichment analysis by using TargetMine [28]. Significantly enriched pathways were protein folding, aminoacyl-tRNA biosynthesis, RNA transport, Wnt signalling, Huntington’s disease/Alzheimer’s disease, synaptic vesicle cycle and Hippo signalling (Table 5). Of note, nuclear shift was found for those proteins involved in RNA transport/processing and Huntington’s disease/Alzheimer’s disease, while cytoplasmic shift for those proteins related to protein folding, aminoacyl-tRNA biosynthesis, Wnt signalling, synaptic vesicle cycle and Hippo signalling pathways (Fig 5).

**Table 5:** Integrated pathway clusters enriched for the differentially localized proteins.

Integrated pathway clusters	Genes	P-value
Protein folding	Cct5, Cct7, Cct8	0.00055
Aminoacyl-tRNA biosynthesis	Farsb, Nars, Txnrd1	0.0018
RNA transport/ Processing of capped intron-containing pre-mRNA	Dhx9, Fmr1, Srsf3, Srsf6, Tra2b	0.0068
Signalling by Wnt	Cltc, Plcb3, Plec, Psmd3, Ruvbl1	0.011
Huntington's disease/ Alzheimer's disease	Atp5b, Atp5d, Cltc, Plcb3, Sod1	0.018
Synaptic vesicle cycle	Cltc, Nsf	0.018
Hippo signalling pathway	Camk2d, Plcb3, Ruvbl1	0.049

### Validation of proteome data

**Fig 6:**
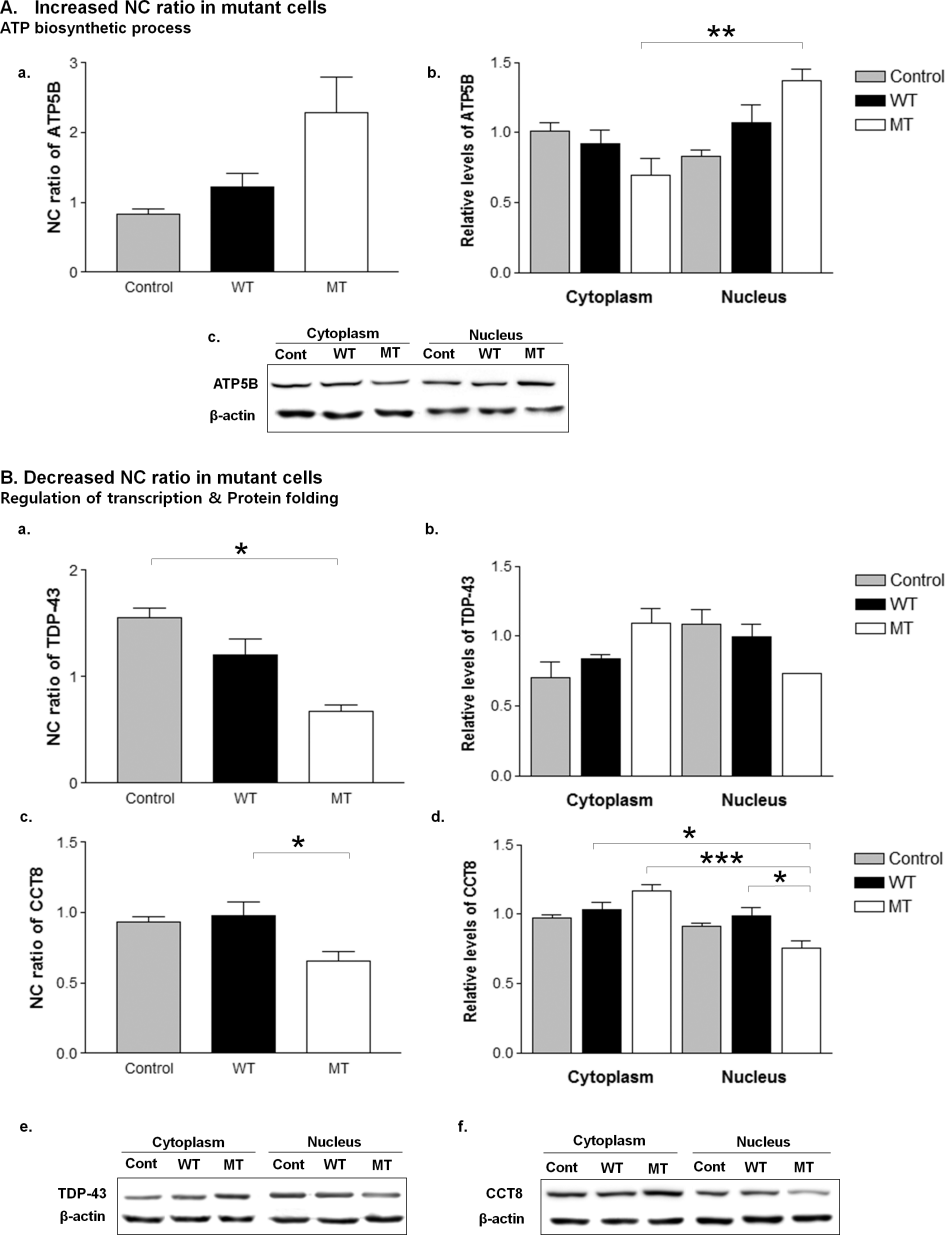
Validation of the proteome data. A subset of differentially localized proteins was validated by western blot. The nuclear shift of ATP5B (ATP biosynthetic process) and the cytoplasmic shifts of TDP-43 (associated with regulation of transcription) and CCT8 (associated with protein folding) were confirmed (A, B). The protein expression level was normalized to β-actin. The data were reported as the mean ± SE. NC ratio, nucleocytoplasmic ratio. *p<0.05, **p<0.01, ***p<0.001, n = 3 per group. Mann-Whitney U test was used to compare the NC ratio, and two-way ANOVA with Tukey’s test was used to compare protein expression levels in the nuclear and cytoplasmic fractions of wild-type and mutant cells. Cont, control; NC, nucleocytoplasmic; MT, mutant cells; WT, wild-type cells.

For validation of the proteome data, we prepared fractionated lysates from two mock transfected control, five wild-type and G93A mutant hSOD1-transfected NSC34 cells, and conducted western blot for a subset of proteins as followings: chaperonin containing TCP1 subunit 5 (CCT5), chaperonin containing TCP1 subunit 7 (CCT7), chaperonin containing TCP1 subunit 8 (CCT8), asparaginyl-tRNA synthetase (NARS), phenylalanyl-tRNA synthetase beta subunit (FARSB), RuvB Like AAA ATPase 1 (RUVBL1), calcium/calmodulin-dependent protein kinase type II subunit beta (CAMK2D), host cell factor 1 (HCF1), cullin-associated NEDD8-dissociated protein 1 (CAND1), TAR DNA-binding protein 43 (TDP-43), ATP synthase subunit beta, mitochondrial (ATP5B), ATP synthase subunit delta, mitochondrial (ATP5D), superoxide dismutase 1 (SOD1), histone H1.1 (HIST1H1A), histone H1.5 (HIST1H1B), histone H1.4 (HIST1H1E). A nuclear shift was confirmed for ATP5B, and a cytoplasmic shift for TDP-43, and CCT8 (Fig 6). Furthermore, the proteins related to nucleosome assembly such as HIST1H1A, HIST1H1B, HIST1H1E were confirmed to be down-regulated in mutant cells (data not shown).

### Transcriptome analysis

**Fig 7:**
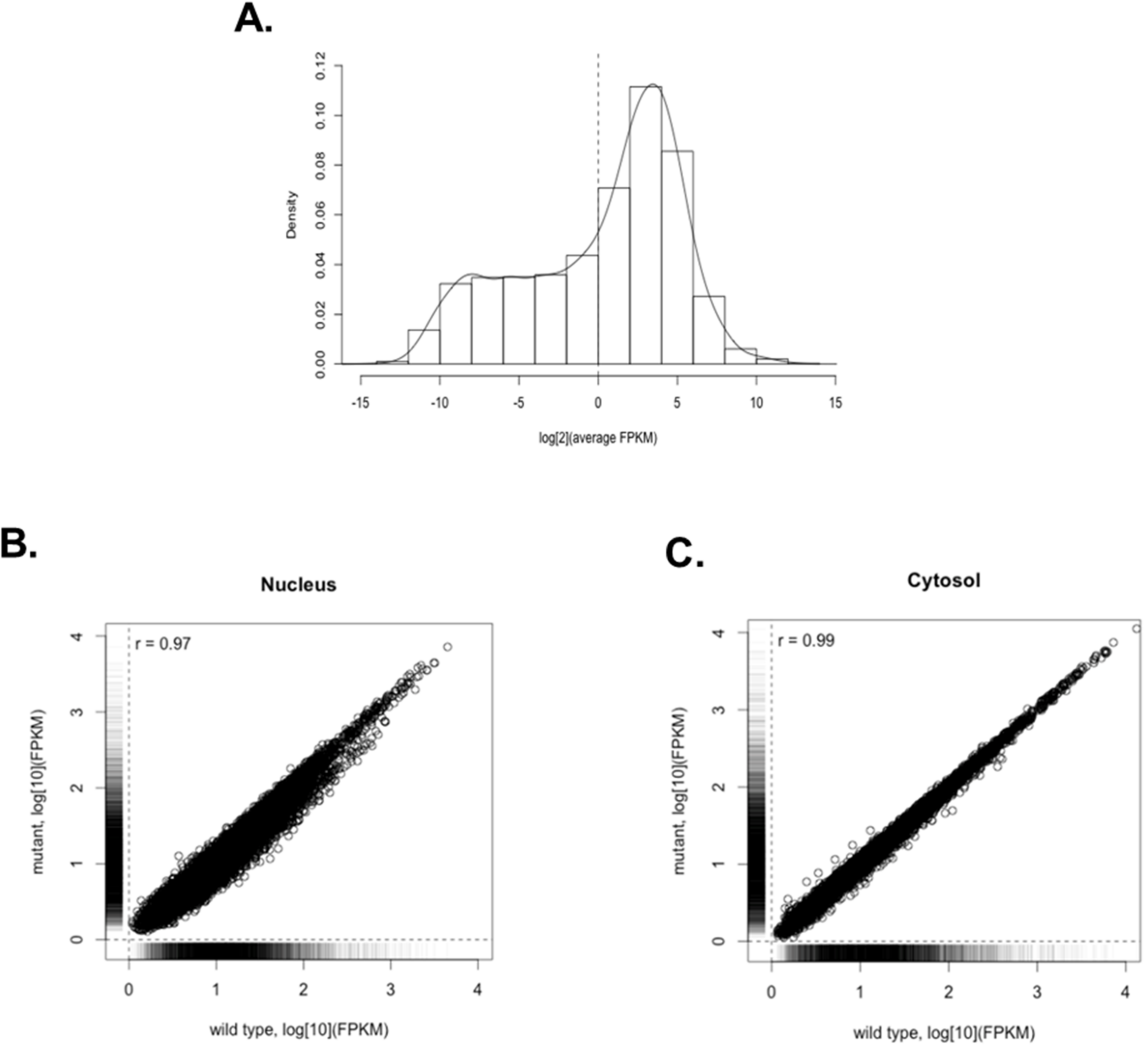
Density plot showing the distribution of average levels of RNA expression (A) and correlations of RNA expression between wild-type and mutant cells in the nucleus (B) and the cytosol (C).

To evaluate the change of nucleocytoplasmic distribution in RNA level, RNA-seq was performed. Total RNA samples from wild-type and G93A mutant hSOD1-expressing NSC34 cells were analysed in triplicates. A distribution of the average expression levels (measured as fragments per kilobase of transcript per million mapped fragments, FPKM) is shown in Fig 7A. Most genes exhibited a normal distribution, and a few genes formed a ‘shoulder’ to left of the distribution. We excluded the very low abundance transcripts with an average FPKM <1 (n = 7231), presumably non-functional, from our subsequent analysis. The absolute transcript levels from wild-type and mutant cells were well correlated in both subcellular compartments (Pearson correlation coefficient r = 0.97 and r = 0.99) (Fig 7B and 7C). These results suggest that mutant (G93A) hSOD1-expressing NSC34 cells primarily retain the characteristics of wild-type cells with regard to gene expression. Only 7 transcripts were significantly up-regulated in the whole extracts of mutant cells compared to wild-type cells, and no transcripts were significantly down-regulated in mutant cells. The nucleocytoplasmic distribution of 9 transcripts was significantly altered in mutant cells: cytoplasmic shift in 5 transcripts and nuclear shift in 4 (Table 6).

**Table 6:** Summary of transcriptome analysis.

Gene symbol	Fold-change
**Up-regulated in mutant cells**	
Syt4	2.24
Pnpla7	2.01
Dbh	2.2
Chrna3	2.02
Fmr1nb	2.13
Armcx2	2.72
Magea8	3.04
**Down-regulated in mutant cells**	
None	
**Cytoplasmic shift in mutant cells**	
A930011O12Rik	1.27
Paxbp1	1.58
D4Wsu53e	1.26
1600012H06Rik	1.31
Prpf38b	1.22
**Nuclear shift in mutant cells**	
5730480H06Rik	1.22
Igsf8	1.23
Git2	1.33
Chrna3	1.83

Fold-change represents the change in the nucleocytoplasmic (NC) expression ratio for cytoplasmic and nuclear shifts.

#### Proteome and transcriptome correlation

**Fig 8:**
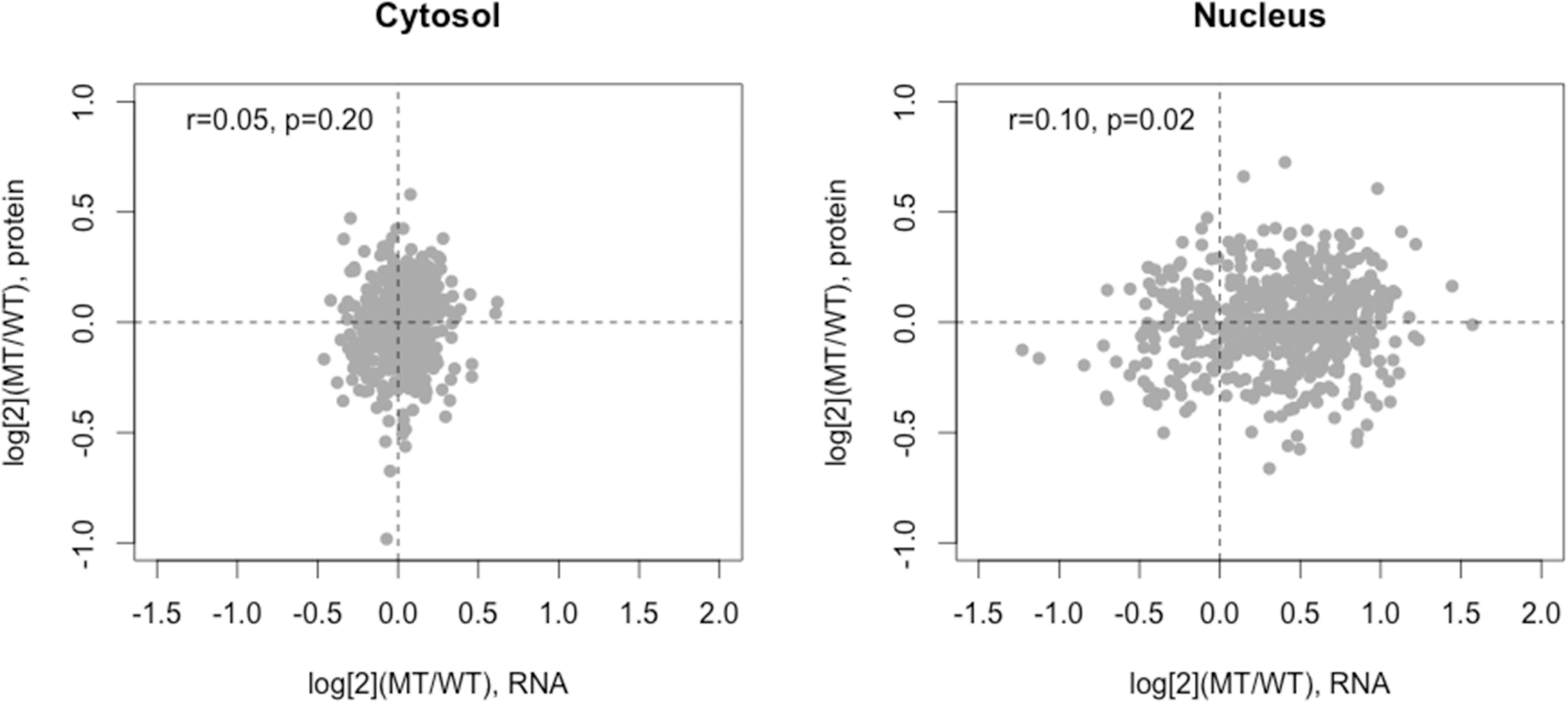
Correlations between the changes in protein and RNA levels in the cytosol (right) and the nucleus (right). The changes in RNA (x axis) and protein (y axis) levels were expressed as log (base 2) ratios of abundance in mutant vs. wild-type cells. WT, wild-type cells; MT, mutant cells. N = 539.

The correlations between the changes in protein abundance and RNA levels were analysed for each subcellular compartment. A weak correlation was found in the nucleus (Pearson correlation coefficient r = 0.1, p = 0.02) but not in the cytoplasm (Fig 8). Of note, RNA in mutant cells was remarkably retained in the nucleus, suggesting either defects in RNA processing or transport to the cytoplasm.

To investigate whether this results from mRNA splicing defects, we quantified intronic and exonic reads in RNA-seq data by using HTseq [26]. As expected, we observed much larger number of intron reads in the nucleus compared to the cytoplasm. The count ratio of intron versus exon reads (referred to as I/E ratio) was 5–6 times larger in the nucleus than in the cytoplasm (S3A Fig). We hypothesized that splicing defects would increase the IE ratio in the presence of G93A mutant hSOD1. Compared to wild-type hSOD1 expressing cells, however, we could not find any statistically significant bias towards increased IE ratio in mutant cells (S3B Fig). Although two-way ANOVA revealed a significant compartment effect (nucleus vs. cytoplasm) effect, there was neither significant genotype effect (wild-type vs. mutant) (p = 0.56) nor genotype × compartment interaction (p = 0.51). Thus, splicing defects, which would lead to increased intron reads, might not account for the nuclear retention of mRNA in mutant cells. Defects in transport or altered metabolism in cytoplasm can be considered as an alternative explanation.

## Discussion

Based on quantitative mass spectrometry and RNA-seq, this study provides a comprehensive unbiased dataset of the nucleocytoplasmic distribution of the proteome and transcriptome in an in vitro model of ALS. Among 634 proteins quantified in both nuclear and cytoplasmic subcellular fractions, we found significant alterations in the nucleocytoplasmic distributions of 37 proteins. The pathway analysis revealed that the proteins shifted towards the nucleus were associated with RNA transport and processing (Dhx9, Fmr1, Srsf3, Srsf6, Tra2b) and that the proteins shifted towards the cytoplasm were associated with protein folding (Cct5, Cct7, Cct8), aminoacyl-tRNA biosynthesis (Farsb, Nars, Txnrd1), synaptic vesicle cycle (Cltc, Nsf), Wnt signalling (Cltc, Plcb3, Plec, Psmd3, Ruvbl1) and Hippo signalling (Camk2d, Plcb3, Ruvbl1) pathways. The transcriptome analysis showed that a high proportion of transcripts were retained within the nucleus in mutant cells, suggesting defects in transport or deficient degradation in cytoplasm. These results suggest that the cytotoxicity of mutant SOD1 may be related to the altered nucleocytoplasmic distributions of proteins and transcripts and possibly to disrupted nucleocytoplasmic transport.

Interestingly, we found that the proteins related to RNA transport and processing were shifted towards the nucleus in the presence of G93A mutant hSOD1. This result was found in parallel with our transcriptome data that showed markedly more abundant nuclear RNAs in mutant cells. In a recent study in expanded GGGGCC fly cells and iPSC-derived neurons of C9orf72-related patients, the ratio of nuclear to cytoplasmic RNA was increased, demonstrating an abnormal increase in nuclear RNAs [6]. The disproportional RNA distribution was considered as evidence of RNA processing/export defects in C9orf72-related disease [6]. Our findings suggest that RNA transport dysfunction might also play a role in causing motor neuron degeneration in SOD1-mediated ALS. Because nuclear retained RNAs were revealed to be spliced by comparing intron reads to exon read in each fraction, RNA processing defect seems less likely to be the cause of nuclear RNA retention in our study.

In this study, we discovered pathological candidates that have not been previously considered. Among these proteins, TDP-43, CCT8 and ATP5B were validated for their altered nucleocytoplasmic distributions in mutant cells. TDP-43 is a predominantly nuclear protein and its redistribution to cytoplasm is known as a key pathological hallmark of ALS. Majority of sporadic and familial ALS have cytoplasmic TDP-43 aggregation [2–4]. However, it still remains controversial whether the TDP43 pathology occurs in SOD1-ALS [29–32]. Our subcellular proteome analysis clearly demonstrated TDP-43 redistribution in the presence of G93A mutant hSOD1, which was validated by western blot and immunofluorescence staining (data not shown). This in line with previous studies that showed TDP-43 redistribution and aggregation in G93A mutant SOD1 mice and familial ALS cases with SOD1 mutation [30, 32]. The role of TDP-43 pathology in SOD1 mutation needs to be further evaluated. Cct8 are members of the chaperonins containing the TCP-1 complex, which are related to protein synthesis, transport and proper folding. Incorrect protein folding leads to protein aggregation, and chaperonins containing the TCP-1 complex were reported to suppress aggregation in a Huntington’s disease model [33]. Chaperonins containing the TCP-1 complex (specifically the subunit of Cct1) bind directly to the huntingtin protein in mutant huntingtin transgenic mice. Suppression of Cct1 resulted in increased huntingtin protein aggregation [34,35]. ALS shares common pathology with Huntington’s disease in that both have cytoplasmic aggregations. Cytoplasmic shifts of Cct8 might indicate a compensatory response to remove the cytoplasmic aggregate. Atp5b, a subunit of the mitochondrial ATP synthase, is normally placed in the inner membrane of the mitochondria. In our experimental protocol of subcellular fractionation, the mitochondria fraction is expected to be mixed in with the cytoplasmic extracts. Relative increments of Atp5b in the nucleus therefore might reflect relative depletion of Atp5b in the mitochondria in mutant cells. A lower expression of ATP synthase and its dysfunction have been described in many neurodegenerative disorders, including Alzheimer’s disease [36]. Dysfunctional mitochondria seemed to change amyloid precursor protein metabolism and enhance the amyloid β-peptide aggregation in the cytoplasm in Alzheimer’s disease [37]. Our study is the first to raise the possible association of chaperonins containing the TCP-1 complex (Cct8) and ATP synthase (Atp5b) with ALS pathology, which warrants investigation in future studies.

In this study, validation with the total cell lysates of mutant cells revealed down-regulation of the proteins associated with nucleosome assembly and phosphate metabolic processes. Nucleosome assembly participates in storing genomic information and regulating DNA-related process such as transcription, repair and replications [38]. While the effects of diminished nucleosome assembly remain unclear, one group recently succeeded in generating nucleosome-depleted paternal pronuclei by deleting maternal histone or associated chaperone HIRA in mouse zygotes [39]. The nucleosome assembly depletion resulted in a loss of nuclear pore complex in the nuclear envelop. This seemed to be related to mislocalization of the nuclear pore complex protein ELYS [39]. Along with these results, our findings might provide additional indirect evidence of nuclear pore complex dysfunction in the presence of G93A mutant hSOD1.

A correlation between the changes in proteome and transcriptome levels in mutant cells was found to be modest only in the nucleus but not in the cytoplasm. This was consistent with previous reports that showed weak or no correlation in diverse species from eukaryotes to yeast [40–43]. Although transcript and protein levels were linked, there are many regulatory processes that weaken their correlation such as translational and post-translational regulation, structural and physiological properties of proteins and degradation rates of proteins and mRNAs.

Large-scale proteomics studies on ALS models are relatively scarce, and direct comparison with other studies would not be straightforward due to differences in disease model (cell line, rodent tissue), experimental design and method for proteome analysis. It is possible, however, to find several changes that are similar with other studies. The most remarkable examples are the proteins involved in defense to oxidative stress [44, 45]—glutathione S-transferase Mu 1 (GSTM1), glutathione S-transferase Pi B (GSTP1), peroxiredoxin 6—and protein folding [46]—protein disulfide isomerase A3 (PDIA3), heat shock 70 kDa protein 8 (HSPA8). Other common pathways include cell death signal transduction [47], intracellular protein trafficking [45], and protein-nucleic acid interactions [48].

There are several limitations to acknowledge in this study. Although we used a high-resolution quantitative proteomic technique, the issues of proteome coverage range, and the risk of false positive identification remain. Indeed, our proteome coverage was low compared to that of transcriptome data. Technical limitations related to the physicochemical properties of proteins (molecular weight, hydrophobicity, coding sequence length, isoelectric points, etc.), and biological process such as posttranscriptional regulation may have influenced the coverage rate. In this regard, we found higher proteome coverage for more abundant transcripts (S4 Fig), and therefore, the proteins quantified in this study were biased as the most abundant proteins. Nevertheless, this study provides the first comprehensive genome-wide dataset of the nucleocytoplasmic distribution of proteins and RNAs in an in vitro model of ALS. The integrated analysis of the nucleocytoplasmic distribution of the proteome and transcriptome revealed multiple candidate pathways including RNA processing/transport and protein synthesis and folding that may be relevant to the pathomechanism of ALS.

## Supporting information

S1 Fig In silico quality check of nuclear-cytoplasmic fractionation. (A) Distribution and organelle membership of marker proteins found in our proteome data. The proteins that are exclusively identified in nuclear fraction were mostly labeled as nuclear organelles such as nucleus, chromatin, and nucleolus. In contrast, the most representative organelles of the proteins exclusively identified in cytoplasmic fraction were proteasome, cytoplasm, and mitochondria. (B) Principal component analysis of the quantified proteins from the wild-type (wt) and mutant (mt) cells are shown. Samples from different subcellular fractions are colour coded (red for cytoplasm, and cyan for nucleus). The x-axis and y-axis are labelled with the first and second principal components accounting for 70% and 12.9% of the total variation, respectively. (B) The distribution of cytoplasmic (left) and nuclear (right) marker proteins across 12 samples (x-axis, triplicates for each group) are presented as their relative abundance and expressed as the z-score (y-axis). Light blue represents the marker proteins corresponding to the subcellular fraction; light grey denotes other proteins. cy, cytoplasmic fraction; nu, nuclear fraction. The marker proteins were obtained from pRoloc’s organelle markers [27].
(TIF)

S2 Fig Bar plot showing the number of genes in the pathways that were significantly enriched for the quantified proteins. The integrated pathway cluster analysis was performed using TargetMine to evaluate their biological function. Significance was set at an adjusted p-value = 0.05.
(TIF)

S3 Fig Comparison of intronic and exonic reads in RNA-seq data. Quantification of reads was performed by using HTseq with an intersection-strict option [26]. (A) There was considerably larger number of intron reads relative to exon reads in nucleus compared to cytoplasm. Even with significant compartment effect (nucleus vs. cytoplasm) on the count ratio of intron versus exon reads (referred to as IE ratio), there was no significant effect of genotype (p = 0.56) or genotype × compartment interaction (p = 0.51). (B) There was no bias towards increased IE ratio in mutant cells. IE ratio, Intron/Exon reads ratio; MT, mutant hSOD1 (G93A)-expressing cells; WT, wild-type hSOD1 expressing cells.
(TIF)

S4 Fig The proteome coverage rate is dependent on the level of transcript abundance. With increasing levels of transcript abundance, the rate of proteome coverage, i.e., the proportion of quantified proteins in mass spectrometry relative to the transcripts identified in RNA-seq, tended to increase.
(TIF)

S1 Table Peptide intensity in cytoplasm fractions of wild-type and mutant cell lines (triplicates). (CSV)

S2 Table Peptide intensity in nucleus fractions of wild-type and mutant cell lines (triplicates). (CSV)

S3 Table Protein abundance after R-rollup. (CSV)
