# Supplementary figures and images for: Altered nucleocytoplasmic proteome and transcriptome distributions in an in vitro model of amyotrophic lateral sclerosis
Source: PLoS One. 2017 Apr 28;12(4):e0176462. doi: 10.1371/journal.pone.0176462 (PMC5409181; doi:10.1371/journal.pone.0176462)

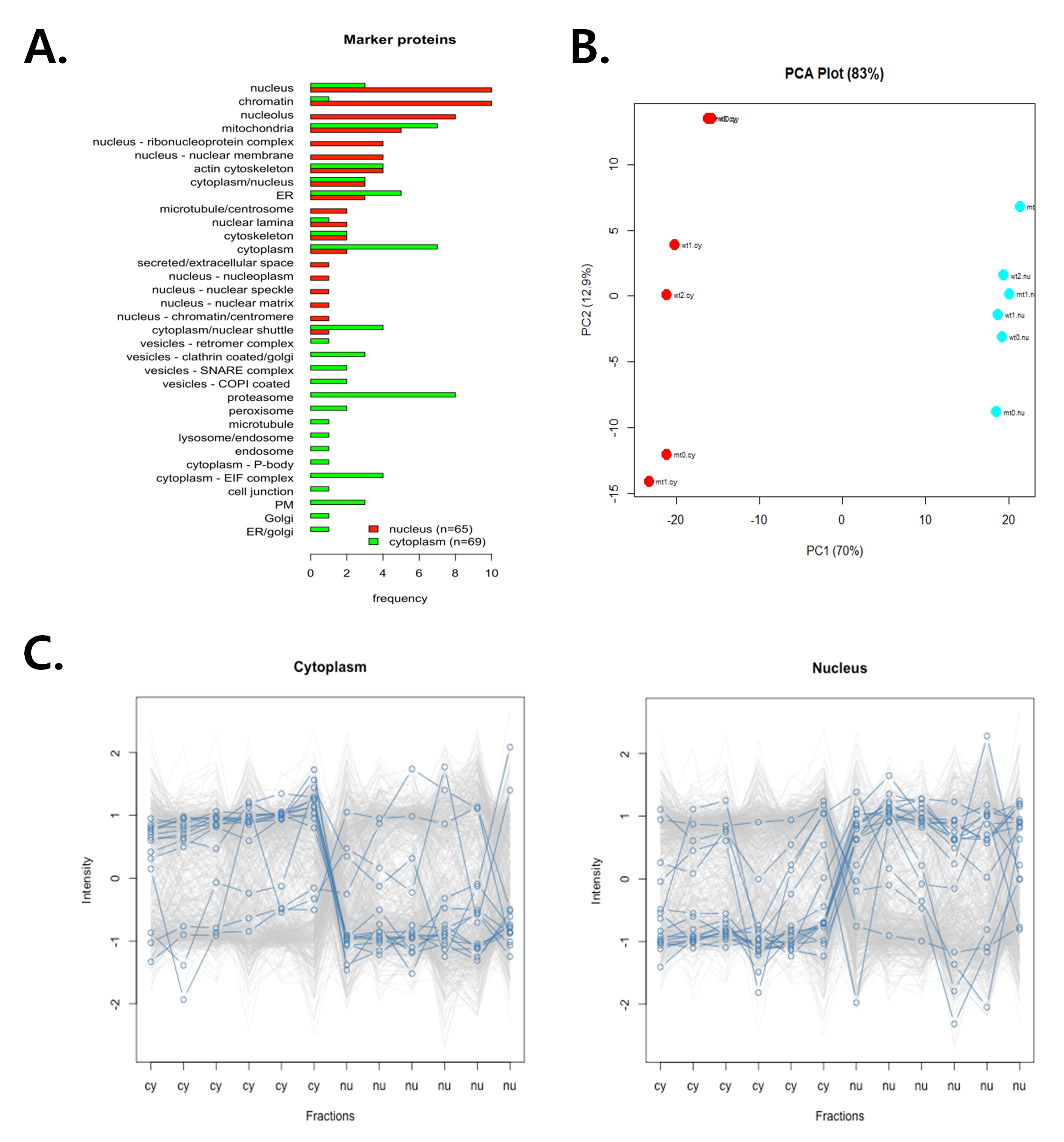

Supplement: S1 Fig — (A) Distribution and organelle membership of marker proteins found in our proteome data. The proteins that are exclusively identified in nuclear fraction were mostly labeled as nuclear organelles such as nucleus, chromatin, and nucleolus. In contrast, the most representative organelles of the proteins exclusively identified in cytoplasmic fraction were proteasome, cytoplasm, and mitochondria. (B) Principal component analysis of the quantified proteins from the wild-type (wt) and mutant (mt) cells are shown. Samples from different subcellular fractions are colour coded (red for cytoplasm, and cyan for nucleus). The x-axis and y-axis are labelled with the first and second principal components accounting for 70% and 12.9% of the total variation, respectively. (B) The distribution of cytoplasmic (left) and nuclear (right) marker proteins across 12 samples (x-axis, triplicates for each group) are presented as their relative abundance and expressed as the z-score (y-axis). Light blue represents the marker proteins corresponding to the subcellular fraction; light grey denotes other proteins. cy, cytoplasmic fraction; nu, nuclear fraction. The marker proteins were obtained from pRoloc’s organelle markers [27]. (TIF) [file pone.0176462.s001.tif]

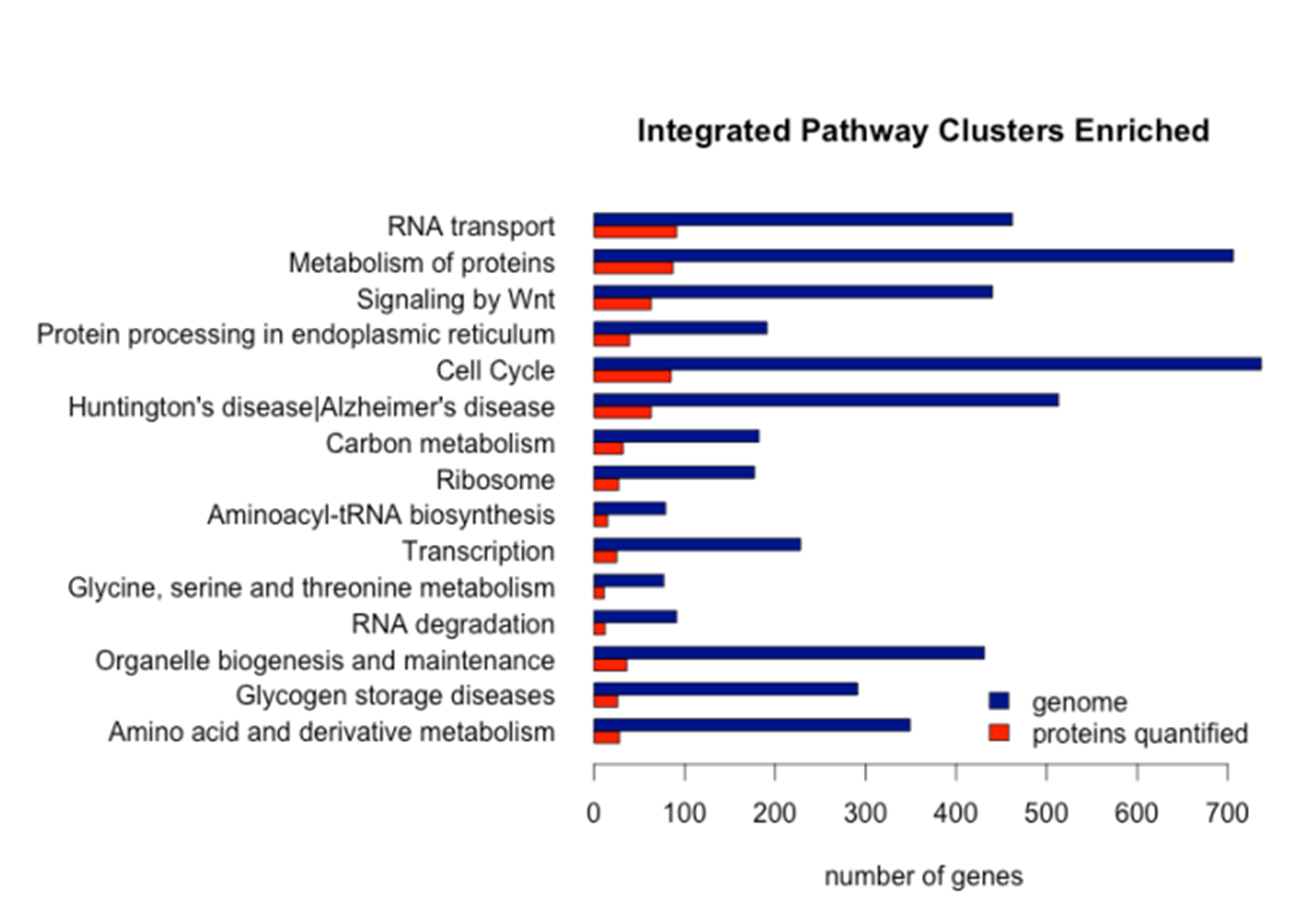

Supplement: S2 Fig — The integrated pathway cluster analysis was performed using TargetMine to evaluate their biological function. Significance was set at an adjusted p-value = 0.05. (TIF) [file pone.0176462.s002.tif]

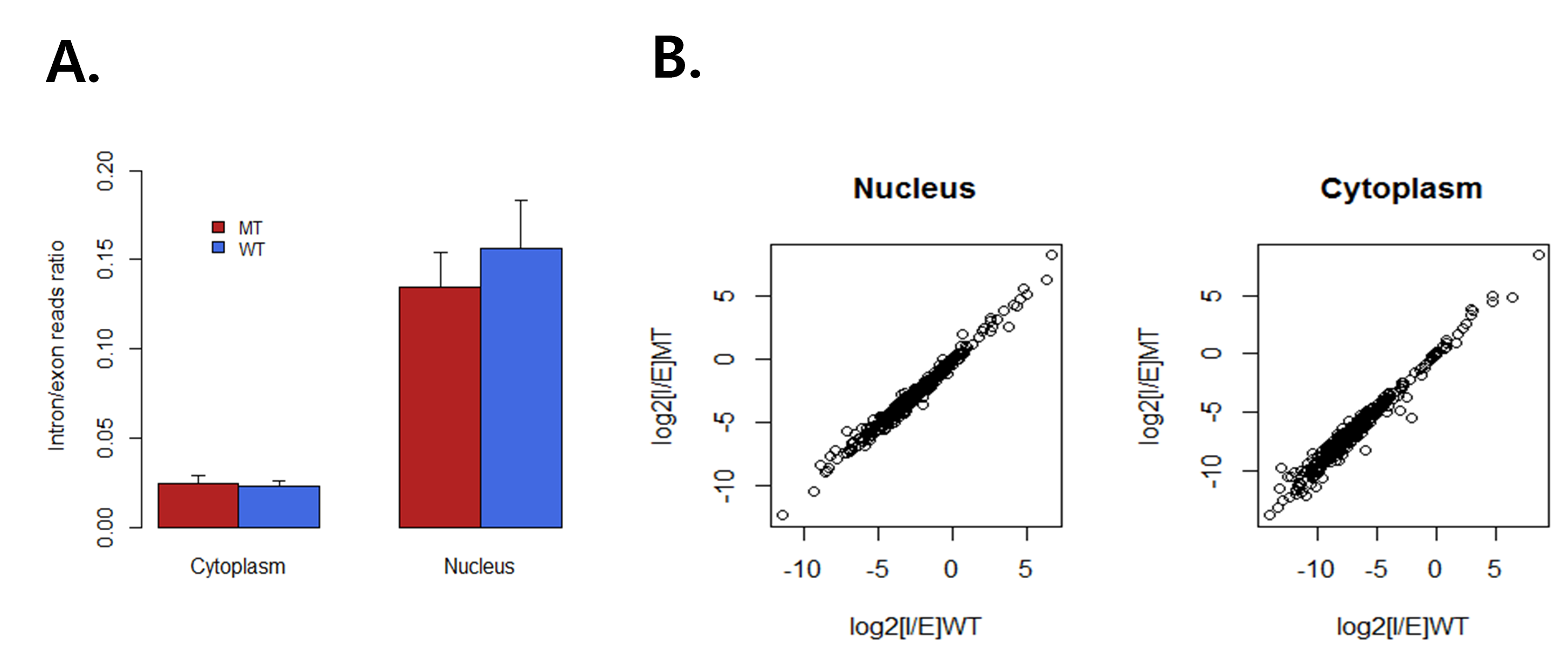

Supplement: S3 Fig — Quantification of reads was performed by using HTseq with an intersection-strict option [26]. (A) There was considerably larger number of intron reads relative to exon reads in nucleus compared to cytoplasm. Even with significant compartment effect (nucleus vs. cytoplasm) on the count ratio of intron versus exon reads (referred to as IE ratio), there was no significant effect of genotype (p = 0.56) or genotype × compartment interaction (p = 0.51). (B) There was no bias towards increased IE ratio in mutant cells. IE ratio, Intron/Exon reads ratio; MT, mutant hSOD1 (G93A)-expressing cells; WT, wild-type hSOD1 expressing cells. (TIF) [file pone.0176462.s003.tif]

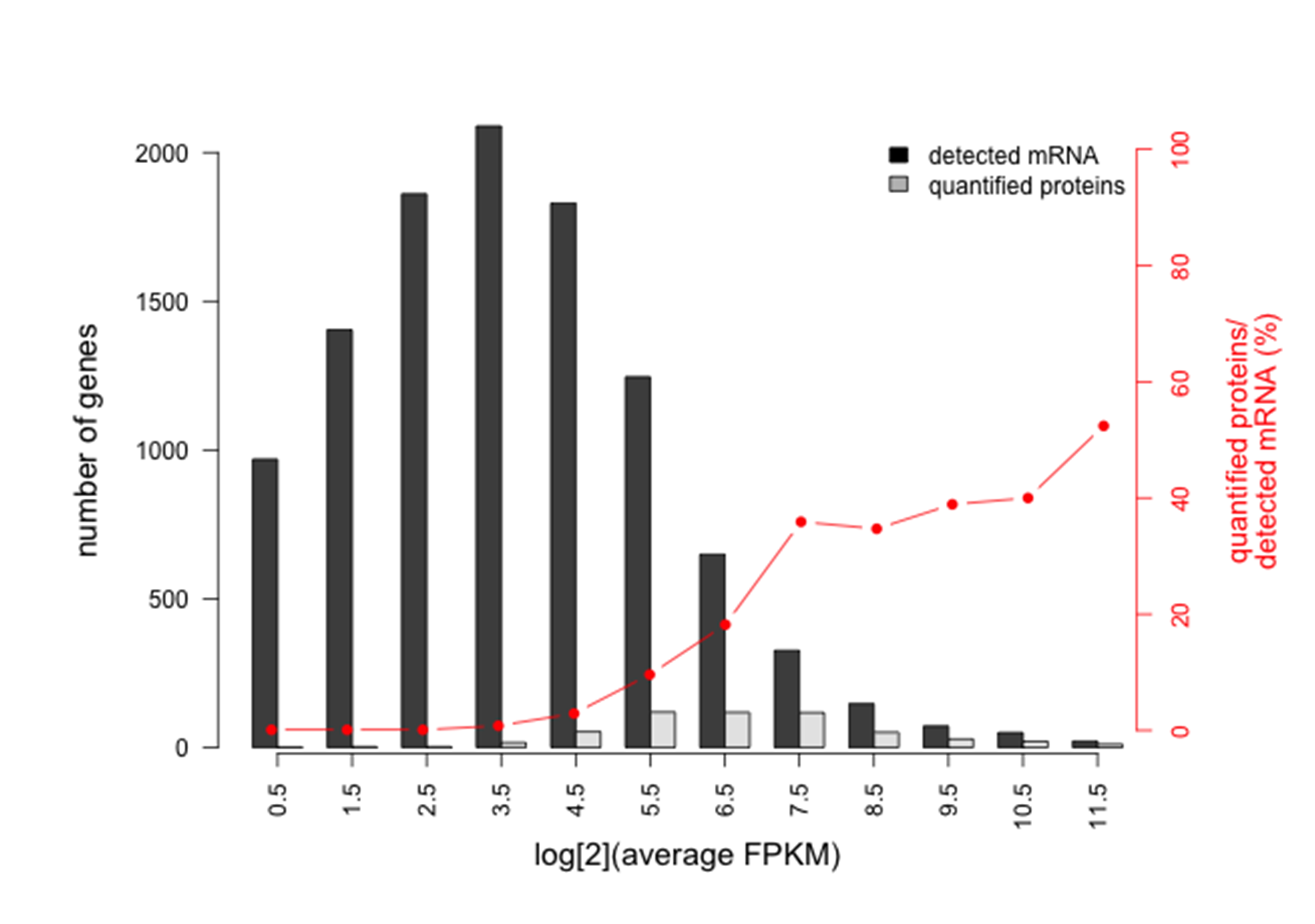

Supplement: S4 Fig — With increasing levels of transcript abundance, the rate of proteome coverage, i.e., the proportion of quantified proteins in mass spectrometry relative to the transcripts identified in RNA-seq, tended to increase. (TIF) [file pone.0176462.s004.tif]
